# Comparison of Nulliparas Undergoing Cesarean Section in First and Second Stages of Labour: A Prospective Study in a Tertiary Teaching Hospital

**DOI:** 10.1155/2011/986506

**Published:** 2011-09-20

**Authors:** Ayhan Sucak, Şevki Çelen, Eren Akbaba, Sunullah Soysal, Ozlem Moraloglu, Nuri Danışman

**Affiliations:** Department of Perinatology, Zekai Tahir Burak Maternity and Research Hospital, Ankara, Turkey

## Abstract

*Objective*. We performed a prospective observational audit study to compare neonatal and maternal outcomes of the primary cesarean sections performed in first stage versus second stage of labour. *Methods*. One thousand three hundred and eighty-nine nullipara women who had undergone cesarean section in a tertiary teaching hospital between February 1, 2009 and January 31, 2010 were included in the study. Primary maternal outcomes of interest were uterine atonia, transfusion requirement, urinary system injury, requirement for hysterectomy, and duration of hospital stay. *Results*. A total of 1389 women underwent cesarean section at this 12 month time period. Of these 1389 cesarean sections, 1271 were in the first stage of the labour and 171 were in the second stage of the labour. Urinary injuries, transfusion requirement, and uterine atonia hysterectomy were significantly more frequent in women who underwent cesarean section in the second stage of the labour compared to women undergoing cesarean section in the first stage of the labour. *Conclusion*. Cesarean section in the second stage of the labour is associated with increased maternal and neonatal morbidities. Special attention is required to the patients undergoing cesarean section in the second stage of the labour.

## 1. Introduction

Cesarean section (CS) is the most commonly performed abdominal operation in women all over the world. Variable rates of CS are reported between and within countries [1–3]. One-fourth of the primary CSs are reported to be performed in the second stage of the labour [4, 5] and are more complicated compared to the ones performed in the first stage. The second stage of the labour can be defined as the time elapsed from full dilatation of the cervix to expulsion of the fetus. The extraction of the impacted head of the fetus from the maternal pelvis constitutes the main difficulty of the CS in the second stage of labour and is associated with increased risks such as hemorrhage, prolonged operation time, and other intraoperative complications [6]. Neonatal mortality and morbidity due to hypoxia and fetal trauma remains to be one of the major issues regarding the CSs performed in the second stages of labour [5, 7]. We present here the comparative prospective data of the maternal and neonatal morbidities in nulliparas undergoing CS in the first and second stage of labour.

## 2. Materials and Methods

One thousand three hundred and eighty-nine nullipara women undergoing CS between February 1, 2009 and January 31, 2010 at Zekai Tahir Burak Maternity Hospital Ankara, were included in the study. Inclusion criteria for the study were nulliparity, fetuses with vertex presentation and gestational age older than 36 weeks of gestation, and having no maternal comorbid disease or no associated obstetric problems such as preeclampsia and diabetes mellitus. Informed consents were obtained from all patients, and the study was approved by the Institutional Review Board.

While 1218 of the 1389 women underwent CS in the first stage of the labour, 171 of the patient cohort underwent CS in the second stage of the labour. The time full cervical dilatation was noted, and manual examination of the decensus was noted in all cases. Cases of the instrumental delivery trials were not included in the study. Because of this reason, success rates of the instrumental delivery attemps were not noted. Maternal age, body mass index, gestational age, augmentation of the labour by oxytocin, and presence of episiotomy before cesarean section were noted peroperatively. Intraoperative uterine atonia, transfusion requirement, urinary system complications, and the requirement of hysterectomy were also noted. The maternal duration of hospital stay, birth weight, and the APGAR score of the newborn at the 5th minute, and the requirement to transport the infant to the neonatal intensive care unit were the postoperative data recorded. Prophylactic antibiotics were administered to all patients after clamping the umbilical cord.

## 3. Statistics

Univariate comparisons were made between the maternal, labour, and postoperative characteristics of the two groups. The two groups were compared for delivery outcomes. Analyses suggested that the responses of the women who went on to have a cesarean section after a failed attempt at instrumental delivery were similar to those of the women who had an immediate cesarean section 15 16. We did not, therefore, perform any subgroup analyses in the cesarean section group.

Univariate analyses were done using logistic regression, followed by multivariate analyses adjusting for potential confounding factors. All reported probability values were 2-sided, and significance was defined a priori as *P* < 0.05. Additional models that were adjusted for maternal body mass index at delivery and duration of the first stage of labour were analyzed when the outcomes were frequent enough to support valid logistic regressions. No adjustments were made for multiple comparisons. SAS software (SAS Institute, Inc, Cary, NC) and LogXact software (Cytel Software Corp, Cambridge, MA) were used for analysis.

## 4. Results

A total of 1389 women had undergone CS in this 12-month period. One hundred and seventy-one (12.3%) of the CS were in the second stage of the labour without a statistical significant difference in ages of the patients who had undergone CS in the first and second stages of the labour. Body mass indexes of the patients in the second stage of the labour (28.1 ± 4.7) were significantly greater than the patients in the first stage of the labour (24.0 ± 3.9) (*P* < 0.05). Gestational ages of the patients were 38.8 ± 1.2 and 40.2 ± 1.7 in first and the second stages of the labour, respectively, with significantly longer duration of pregnancy in the latter group (*P* < 0.05). Three (1.8%) women who had undergone CS in the second stage had required an episiothomy before switching to CS due to failure of labour.  Table 1 shows the preoperative characteristics of the women in both groups.

Nine (%5.3) of the women undergoing CS in the second stage of the labour had uterine atonia significantly more than the 38 (%3.1) women who underwent CS in the first stage of the labour. Atonia and other causes of hemorrhage resulted in a significantly higher transfusion requirement in women undergoing CS in the second stage of labour ((22 4.1% versus 7 1.8%) *P* < 0.05). Inevitable hysterectomy in five women (%1.2) in the second stage of the labour was significantly more frequent compared to the 2 (%0.4) women in the first stage of the labour. Urinary system injury was also more frequent in the patients undergoing CS in the second stage of labour (7 (4.2%) versus 15 (1.2%), resp. (*P* < 0.05)). All the urinary system injuries were confined to the bladder without any ureteral injuries in both groups. The extent of the injuries were up to serosal defect formation, and no whole layer cystostomy was noted in both groups. The most common bladder injury was the ones due to traction resulting in hematuria.  Table 2 shows the intraoperative complications of the cesarean section in the two groups.

Women undergoing CS in the second stage of the labour required significantly longer hospital stay (3.3 ± 0.8 days) compared to the patients requiring CS in the first stage of the labour (2.2 ± 0.4 days) (*P* < 0.05).

Birth weights of the neonates born to mothers who had undergone CS in the second stage of the labour (3780 ± 635 g) was heavier than the neonates (3310 ± 455 g) born to mothers who had undergone CS in the first stage of the labour (*P* < 0.05). APGAR score less than 4 at 5th minute was more frequent (1.1%) in the newborns of the mothers operated in the second stage of the labour compared to the patients operated in the first stage (0.6%), however without statistical significance (*P* > 0.05). Fifty of the newborns (4.1%) born to mothers operated in the first stage of the labour were transported to the newborn intensive care unit, compared to 13 (7.6%) newborns born to mothers operated in the second stage of the labour. This result was statistically significant (*P* < 0.05) (Table 3).

## 5. Discussion

We found that primary CS in the second stage of labour in nullipara comprised 12.3% of the total primary Cesarean sections. Relatively lower rate of CS performed in the second stage of labour in the presented study compared to the previously published reports [4] can be explained by a higher rate of elective CS in our institution. The body mass index of the women requiring CS at the second stage of labour was significantly higher, suggesting that obesity is not only an operative but an obstetric risk as well. In general the maternal morbidities can be attributed to the difficulty in handling the fetus impacted to the maternal pelvis. Allen et al. had compared the maternal and neonatal morbidity of the CSs in the first and second stages of the labour in a similar but retrospective study [8]. In this retrospective study women undergoing cesarean section had a 2,6 times risk of maternal morbidity compared to women operated in the first stage of the labour. However, they did not find any difference in the rates of blood transfusion requirement, hysterectomy, or postpartum hemorrhage. Gestational ages of the women requiring CS in the second stage of labour were significantly higher indicating a more advanced stage of pregnancy, and this remains to be explained.

The present study demonstrates that the CSs performed in the second stage of the labour have significantly higher maternal and neonatal morbidity. The maternal morbidities can be attributed to the difficulty in handling the fetus impacted to the maternal pelvis. The unfavourable neonatal outcomes are probably due to the prolongation of the labour which leads to an inevitable result, hypoxia. Previous studies had also shown adverse outcomes of the neonates when the second stage of the labour is longer than the normal [9–12]. Similar to our results they have demonstrated an increased risk of perinatal asphyxia. Cebekulu and Buchmann in South Africa randomised 39 patients undergoing cesarean section in the first stage of the labour and 39 in the second stage of the labour and have also concluded that CSs in the second stage of the labour causes more maternal and neonatal morbidity [13]. A multicenter study conducted in 13 university centers also revealed that cesarean deliveries in the second stage of the labour showed marginally increased maternal but not neonatal morbidity [14]. We have demonstrated that bladder injury was increased about 4 times in the CS performed in the second stage of the labour which suggests these operations are technically more difficult. Development of uterine atony and the requirement for hysterectomy in cases of severe hemorrhage are also found to be more frequent in the CSs performed in the second stage of the labour and can be attributed to longer labour resulting in uterine fatigue. Increased frequency of uterine atony and emergency hysterectomy in patients requiring CS at the second stage of labour seems to be the main cause of increased transfusion requirement in this group. 

There are controversies regarding the fetal outcome of the CS performed in the second stage of labour, while some previous studies fail to demonstrate an increased fetal complication rate [8, 14, 15]. In contrast to some other studies including the current study, the risk of CS in the second stage of labour is not confined to the mother and has adverse prognostic impact on fetal outcome as well [9–11]. 

In this respect women requiring CS in the second stage of labour should be handled and operated by experienced obstetric surgeons. One can think that trial of labour in the second stage of the delivery is not mentioned in this study, but it is clear that cases studied in this study are the ones who were not successful in instrumental delivery attempts. Predicting patients who would require CS in the earlier phases of labour might reduce the maternal and neonatal morbidity. Higher birth weight of the neonates born to women operated in the second stage of labour might suggest that the decision of CS at least in women with big fetuses should be made in advance. One other precaution to reduce fetal morbidity might be to alarm the neonatologists in every case of CS performed in the second stage of labour.

In conclusion, this study suggests that women undergoing cesarean section in the second stage of the labour have increased maternal and fetal morbidity and require special care. The authors recommend that methods to predict patients who would later require CS with the progress of labour should be developed by obsterticians to reduce the rate of CSs in the second stage of labour which does not seem to be innocent. However, it should not increase the suspicious clinical approach to women who can have a normal birth.

## Figures and Tables

**Table 1:** Patient characteristics.

	First stage of the labour	Second stage of the labour	*P*
	*n* = 1218	*n* = 171	
Maternal age	22.3 ± 4.8	23.1 ± 5.2	NS
BMI	24.0 ± 3.9	28.1 ± 4.7	*P* < 0.05
Gestational age (weeks)	38.8 ± 1.2	40.2 ± 1.7	*P* < 0.05
Use of oxytocin	438 (%36)	92 (%54)	*P* < 0.05
Episiotomy	0 (%0)	3 (%1.8)	

NS: not significant.

**Table 2:** Intraoperative findings and complications of cesarean sections in the first and second stages of the labour.

	First stage of the labour	Second stage of the labour	*P *
	(*n* = 1218)	(*n* = 171)	
Uterine atonia	38 (%3.1)	9 (%5.3)	*P* < 0.05
Transfusion requirement	22 (%1.8)	7 (%4.1)	*P* < 0.05
Urinary system injury	15 (%1.2)	7 (%4.1)	*P* < 0.05
Hysterectomy	5 (%0.4)	2 (%1.2)	*P* < 0.05

**Table 3:** Neonatal complications.

	First stage of the labour	Second stage of the labour	Statistical significance
	*n* = 1218	*n* = 171	
APGAR < 4 at the 5th minute	7 (%0.6)	2 (%1.1)	NS
Birth weight	3310 ± 455	3780 ± 635	*P* < 0.05
Requirement for NICU	50 (%4.1)	13 (%7.6)	*P* < 0.05

NS: not significant.
